# Traumatic diaphragmatic rupture successfully managed in 4-year-old patient: Case report and literature review

**DOI:** 10.1016/j.ijscr.2020.06.026

**Published:** 2020-06-11

**Authors:** Adel Elkbuli, Evander Meneses, Aaron Shepherd, Mark McKenney, Dessy Boneva

**Affiliations:** a Department of Surgery, Kendall Regional Medical Center, Miami, FL, USA; b University of South Florida, Tampa, FL, USA

**Keywords:** Pediatric trauma, Motor vehicle collisions, Traumatic diaphragmatic hernia, Trauma outcomes

## Abstract

**Introduction:**

Traumatic diaphragmatic ruptures are rare injuries in the pediatric population that can present with dyspnea, abdominal pain, or even be asymptomatic.

**Case presentation:**

A 4-year-old boy presents to our Level 1 Pediatric Trauma Center after being hit by a car. He presented awake and alert but with low oxygen saturations and chief complaint of left sided abdominal and chest pain. The initial chest radiograph showed an elevated left diaphragm and bilateral opacities. A laparoscopic approach confirmed the diagnosis of a diaphragm rupture and an open approach allowed for the diaphragm repair, after returning the stomach, colon and spleen back to the abdominal cavity. He had an uneventful recovery and was discharged home on postoperative day seven.

**Discussion:**

Traumatic diaphragmatic hernia continues to be a difficult diagnosis to make without a high index of suspicion with variable time to diagnosis. This has been attributed to nonspecific clinical presentation and low incidence of the condition. Chest radiograph has been suggested to be an appropriate initial imaging modality with computed tomography as an accurate adjunct. Right sided diaphragm hernias, although less common, still occur and are often misdiagnosed. Recovery without significant morbidity after definitive surgical treatment with laparotomy is common.

**Conclusion:**

Consider a traumatic diaphragmatic hernia in a blunt pediatric trauma patient with abdominal pain and dyspnea once other life-threatening injuries have been ruled out. This is an easily missed injury that can lead to significant morbidity and mortality if not identified early in the patient’s hospital course.

## Highlights

• Presentation of a left sided traumatic diaphragmatic rupture following a blunt trauma in a pediatric patient.
• Rapid assessment and identification of a traumatic diaphragmatic hernia with prompt repair.
• Uncomplicated postoperative course and hospital discharge following traumatic diaphragmatic hernia excellent outcomes.

## 1 Introduction

Traumatic diaphragmatic rupture (TDR) is a rare entity in the pediatric trauma population. Often an overlooked injury, the pediatric trauma patient can suffer significant morbidity and possibly mortality if undiagnosed. TDRs tend to coexist with other injuries that are immediately life threatening. While traditionally there is a selective non-operative approach for many injuries of intra-abdominal solid organs, TDR requires immediate operation. The associated injuries can carry a high rate of morbidity and mortality [[1], [2], [3], [4]]. In order to provide the proper care for this critical injury, the provider must have a high index of suspicion, as the presentation is often not straightforward and missing this injury can cause devastating sequelae to the patient.

The prevalence of TDRs is 0.07 % of all pediatric trauma patients and are more commonly seen in males. When looking specifically at blunt abdominal trauma, the incidence increases to 2.95 % [[4], [5], [6]]. A minority of TDR cases are the result of penetrating abdominal injury while the vast majority are caused by blunt abdominal trauma and are most commonly due to motor vehicle collisions (MVCs) [7]. The most common symptoms that patients report are dyspnea (86 %) and abdominal pain (13 %). There is often decreased breath sounds on the affected side (73 %) [8].

The pathophysiology in blunt injury is not completely understood, however it is hypothesized that an increased intraabdominal pressure following a blunt mechanism creates a high-pressure gradient between the chest and abdomen to cause the rupture which can further cause abdominal visceral intrathoracic herniation [9]. The most common organ to herniate through the defect is the stomach [3].

We present a case of a pediatric patient who presented to our Level 1 Pediatric Trauma Center after being hit by a car. He suffered a TDR with herniation of stomach, colon, and spleen into the thoracic cavity for which he was taken emergently to the operating room. He underwent diagnostic laparoscopy that was converted to an exploratory laparotomy, reduction of herniated abdominal viscera and repair of the left diaphragmatic rupture. We also reviewed the current literature for this rare entity. Further research is needed to provide surgeons with best practice methods in order to provide optimal treatment for these patients. This case was reported in line with SCARE criteria [10].

## 2 Case presentation

A 4-year-old boy presented to our Level 1 Trauma Center after being hit by a car in the parking lot where he was playing. The patient arrived to the Emergency Department awake and alert, however in respiratory distress. Primary survey revealed that his airway was intact. He had diminished breath sounds on the left side. GCS was 15. External injuries included an abrasion to the left hip. Blood pressure was 101/66, heart rate 174, and his oxygen saturation was in mid 80 percent range. FAST was negative. Patient was complaining of abdominal pain and chest pain rated 10 out of 10 in severity. He also had labored breathing.

**Fig. 1:**
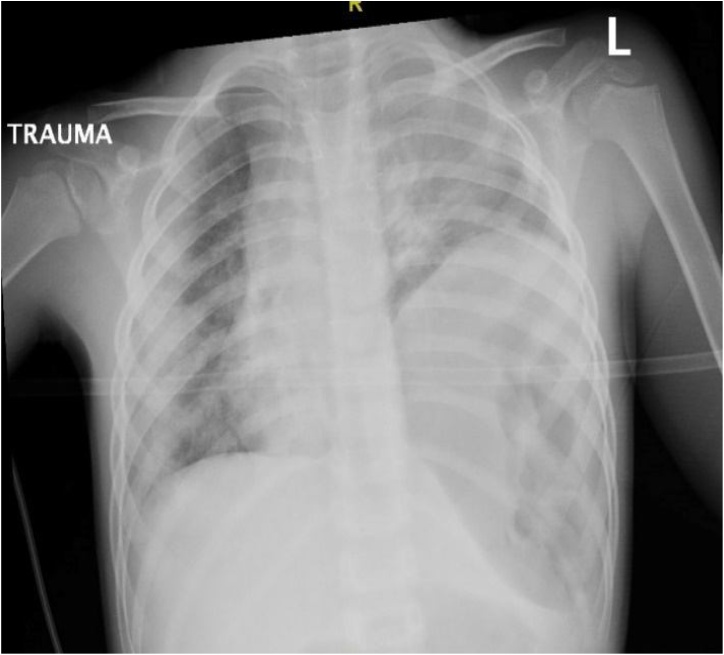
Pre-Operative Chest radiograph.

Given the respiratory status, patient was intubated in the trauma bay. A chest radiograph was taken to evaluate for any life-threatening thoracic injuries as well as to confirm the position of the endotracheal tube (Fig. 1). The chest radiograph revealed an elevated left hemi-diaphragm with patchy parenchymal opacities bilaterally. Initial labs were significant for leukocytosis of 15,700 (normal 3,600−11,000), normal creatinine 0.53 (normal 0.12–1.06), elevated AST 1,459 (normal 10–60), ALT 941 (normal 10–60), amylase 160 (normal 25–115), lipase 941 (normal 23–300), and lactate dehydrogenase 2,669 (normal 84–246). Calculated Blunt Abdominal Trauma in Children (BATiC) score was 9, indicating risk for abdominal injury.

Due to initial findings in the trauma bay, the diagnosis of rupture of the left diaphragm was strongly considered and a decision was made to take the patient to the operating room. Initially, he underwent a diagnostic laparoscopy. A large posterior traumatic diaphragmatic rupture with herniation of stomach, colon and spleen was identified on laparoscopy and so the decision was made to convert to an exploratory laparotomy. The patient then underwent reduction of herniated contents, and repair of large left diaphragmatic rent. The diaphragmatic defect was closed with a running PDS suture with the pneumothorax suctioned out using a red rubber catheter prior to complete closure.

**Fig. 2:**
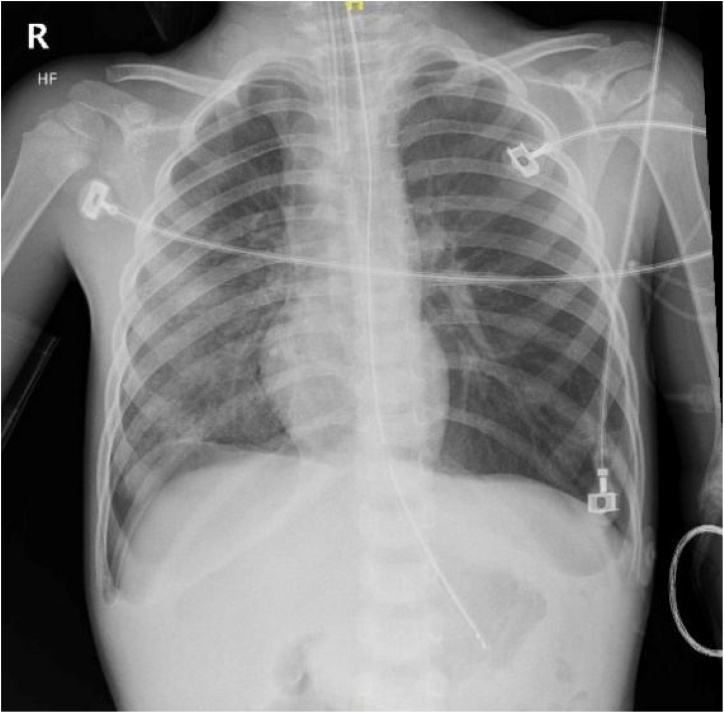
Post-Operative Day 1 Chest radiograph.

On postoperative day one, physical exam revealed no respiratory distress, rales or wheezing, and breath sounds were clear bilaterally (Fig. 2). In addition, his laboratory results normalized, except for the liver function tests, amylase, and lipase, which were improving. His rapid shallow breathing index (RSBI) was less than 105 and he was extubated to nasal cannula oxygen, which was later weaned off. The patient had an uncomplicated course from postoperative day two through six. He was kept until he tolerated a diet and his liver function tests were normalizing. On postoperative day seven, patient was stable for discharge home with laboratory results having normalized. He followed up in the outpatient clinic two weeks later and he was doing well without any respiratory issues.

## 3 Discussion

Our patient presented with significant chest, abdominal pain, and respiratory distress following blunt abdominal trauma after being hit by a car. The respiratory distress led to intubation in the trauma bay. His BATiC score was 9 which proved to be a useful adjunct to diagnosing the abdominal trauma [11,12]. Although the FAST was negative, ultrasound is not a sensitive test to detect diaphragm rupture [13]. The patient’s underlying injury was quickly surmised through the use of a chest radiograph which led to rapid definitive treatment through emergency laparotomy. The trauma surgeon had a high index of suspicion for TDH in this patient, which facilitated the patient’s swift treatment. The quick identification and response led to positive outcomes in this patient with extubation on post operative day 1 and discharge on day 7. In the trauma bay, it was determined that the chest radiograph was sufficient to recognize the high likelihood of TDR and the necessity for rapid surgical intervention due to patient hemodynamic instability. However, computed tomography (CT) may have provided additional information about the patient anatomy and degree of diaphragmatic injury.

The incidence of TDH in children is very low, however; this phenomenon occurs more common than in the adult population [14]. A possible explanation for this occurrence has been Rance et al., which attributes differences in pediatric anatomy such as the thin abdominal wall and more elastic thoracic cage causing an increased propensity to develop diaphragmatic rupture and subsequent herniation [15]. A key factor when managing cases of suspected TDH is the time to diagnosis. The risk of bowel strangulation, respiratory distress, and concealment of more major injuries are serious complications that may arise with delayed diagnosis [16]. A study by Al-Salem found in adults that the time to diagnosis varied greatly, from 3 h to over a week [17]. A literature review regarding TDH in the pediatric population also found that the time to diagnosis was variable, but additionally discovered that diagnosis within the first 24 h only occurred in 58.4 % of patients, which may have led to delayed interventions [18]. Additionally, a study performed in Egypt on adults found that only 40 % of their patients were diagnosed prior to surgical intervention [16]. One likely factor contributing is the lack of symptoms that are specific to TDH. Most patients present with varying degrees of respiratory symptoms and abdominal pain, which could be attributed to several possible etiologies when managing a trauma case [18,19]. This heterogeneity in clinical presentation has been related to the difference in degree of diaphragmatic rupture and bowel herniation [17]. The exception in this case would be bowel sounds present in the hemithorax however this finding is rare and was not discovered in our patient. Another factor that may contribute to the delay in diagnosis is the very low incidence of this condition.

In the literature, the chest radiograph has been described as the best primary test for suspected TDH and the pathognomonic finding of bowel within the thorax has been shown to be found in approximately 85 % of patients [16]. CT is another useful imaging modality for the diagnosis of TDH. CT has been shown to be very sensitive for the diagnosis of acute TDH but less so for the diagnosis of chronic TDH [19,20]. MRI has also been described as a helpful imaging adjunct but as it is generally a time-intensive modality, it may have less utility in situations of acute trauma [19,20]. Left-sided TDH is more common than right-sided TDH, which has been credited to protective effects from the right kidney and liver, however there have been cases of right sided TDH and it is hypothesized that this area is often missed during evaluation [21].

Surgical intervention is the definitive treatment for pediatric TDH. The vast majority of repairs are done through a laparotomy with the use of thoracotomy rarely in special situations such as bilateral TDH [16]. The overall mortality from pediatric TDH is considered to be approximately 9.5 % [16] however, if there are no other significant injuries the mortality rate can be much lower and many patients are discharged without major complications [5,16,19,22]. The association of other lacerations such as splenic, bowel, or lung, in addition to diaphragmatic injury has been described in several studies [5,19,23]. Some of the reported post-operative complications include pancreatitis and pneumonia [16,19]. It has been shown that there is wide variety of time to diagnosis and therefore a delay in definitive treatment for traumatic diaphragmatic ruptures. If the diagnosis remains in doubt or equivocal and a high index of suspicion still remains, multi-detector CT can be used to further evaluate for this injury with a sensitivity of 78 % for left sided injuries and specificity of 100 % [12].

## 4 Conclusion

Traumatic diaphragmatic injuries in the pediatric population are a rare entity and a high index of suspicion is needed in order to accurately diagnose these types of injuries in the setting of negative imaging. Late or missed diagnosis is associated with significant increase in morbidity and mortality. Time to diagnosis can be variable depending on the severity of injury and if the injury is apparent on imaging. Chest radiograph has been well described as helpful on initial imaging. If negative, a multi-detector computed tomography should be considered in order to achieve avoid missed injuries.

## Declaration of Competing Interest

None.

## Funding

None.

## Ethical approval

This report was conducted in compliance with ethical standards. Informed written consent has been obtained and all identifying information is omitted.

## Consent

Informed written consent has been obtained and all identifying information is omitted.

## Author contribution

AE, EM, AS, DB, MM Conception of study, acquisition of data, analysis and interpretation of data, drafting the article, and revision of article. DB, MM – Management of case

AE, EM, AS, DB, MM – Approval of the final version for submission.

## Registration of research studies

This is a case report study.

## Guarantor

Dessy Boneva.

Mark McKenney.

## Provenance and peer review

Not commissioned, externally peer-reviewed
